# Contextual dimensions for cache replacement schemes in information-centric networks: a systematic review

**DOI:** 10.7717/peerj-cs.418

**Published:** 2021-03-11

**Authors:** Stéfani Pires, Artur Ziviani, Leobino N. Sampaio

**Affiliations:** 1 Department of Computer Science, Federal University of Bahia (UFBA), Salvador, Brazil; 2 Federal Institute of Bahia (IFBA), Salvador, Brazil; 3 National Laboratory for Scientific Computing (LNCC), Petrópolis, Brazil

**Keywords:** Information-centric network, Cache replacement policy, Context awareness

## Abstract

In recent years, information-centric networks (ICNs) have gained attention from the research and industry communities as an efficient and reliable content distribution network paradigm, especially to address content-centric and bandwidth-needed applications together with the heterogeneous requirements of emergent networks, such as the Internet of Things (IoT), Vehicular Ad-hoc NETwork (VANET) and Mobile Edge Computing (MEC). In-network caching is an essential part of ICN architecture design, and the performance of the overall network relies on caching policy efficiency. Therefore, a large number of cache replacement strategies have been proposed to suit the needs of different networks. The literature extensively presents studies on the performance of the replacement schemes in different contexts. The evaluations may present different variations of context characteristics leading to different impacts on the performance of the policies or different results of most suitable policies. Conversely, there is a lack of research efforts to understand how the context characteristics influence policy performance. In this direction, we conducted an extensive study of the ICN literature through a Systematic Literature Review (SLR) process to map reported evidence of different aspects of context regarding the cache replacement schemes. Our main findings contribute to the understanding of what is a context from the perspective of cache replacement policies and the context characteristics that influence cache behavior. We also provide a helpful classification of policies based on context dimensions used to determine the relevance of contents. Further, we contribute with a set of cache-enabled networks and their respective context characteristics that enhance the cache eviction process.

## Introduction

The Internet architecture was originally designed in a host-centric paradigm to support end-to-end communication. This model struggles to face key communication requirements of modern network applications such as high content distribution, node’s mobility, and network scalability. Therefore, researchers have devoted effort studying future Internet architectures as alternatives to the IP’s host-centric model. The current practice moves forward to a more content-centric approach since the massive Internet usage is to disseminate content regardless of its location. Information-centric networking (ICN) (Ahlgren et al., 2012) is one of such initiatives. ICN is a content-centric network communication model that stand out as potential candidate to substitute the current TCP/IP model (Rahman et al., 2020). It consists of a receiver-driven networking model that focuses on the distribution and retrieval of contents through a publish-subscribe paradigm. In ICNs, a content request is based on the content’s name, not on its location, such as the content provider’s IP address. Contents should have unique names, and any network node with the content can respond to the request. To this end, ICN replicates content in a distributed way in Cache-enabled Routers (CRs) over the network that are located closer to the user. Therefore, delivering the closest content copies to the user saves communication resources, thus reducing network congestion, server loads, and access latency while providing better Quality of Service (QoS) and Quality of Experience (QoE) levels. In addition, beyond the benefits of in-network caching, decoupling the content delivery process from the content location brings native support to mobility and multicast packet forwarding. Content-Centric Networking (CCN) and its successor Named-Data Networking (NDN) (Zhang et al., 2010) are examples of initiatives implementing ICN concepts.

In general, any network device can potentially work as a CR with a Content Store (CS) data structure to implement the cache service. The performance of CS plays a vital role in the overall packet forwarding engine to guarantee high-speed packet processing of ICN architectures. According to Pan, Huang & Li (2017), Pan et al. (2019), the performance bottleneck of the packet forwarding systems relies on CS operation and should be the focus of ICN optimization strategies. This way, ICN-based initiatives strongly rely on cache replacement policies to manage the CS and keep relevant content available to the users. Least Recently Used (LRU) and Least Frequently Used (LFU) (Ioannou & Weber, 2016) are examples of policies used in ICNs.

The current literature presents a massive number of performance evaluations for cache replacement policies comparing different policies concerning different network contexts. A network context refers to a network type—for example, Edge networks, Internet of Things (IoT) networks, or Vehicular Ad-hoc NETworks (VANETs)—instantiated with particular characteristics for a given purpose. A network context thus brings up a broader view that encompasses characteristics regarding the network type and other entities related to network performance (e.g., user habits while using the network). Each performance evaluation may present distinct variations in the context characteristics, as well as different impacts on policy performances, including changes in performance rank. The variance of results indicates that the policies’ performance tends to vary according to the context’s characteristics, and the process of choosing the suitable policies should consider the context in which the caches operate.

Given the above, several works incorporated the adaptation of policies according to some context. For instance, Beck et al. (2017) proposed a rule-based stream reasoning technique to allow CCN routers to dynamically switch between existing cache replacement policies to adapt to network traffic changes. Moreover, Moon et al. (2016) presented a cache management scheme for wireless NDNs, in which common access points (APs) and user devices attached to the APs have available cache capacity. The authors advocated that each device can choose to work with a different cache replacement policy to improve network performance. In addition to that, Charpinel et al. (2016) proposed a Software-Defined Networking (SDN) approach to provide programable cache replacement algorithms. The replacement algorithms are defined in a control plane. Meanwhile, a CCN controller can modify the replacement schemes dynamically and allocate different strategies for each node. Finally, Pacifici & Dán (2013) proposed autonomous caching in peering ISPs for collaborative deciding their replacement policies.

Although studies recognize the need to adopt policies according to the network context, the choice itself of a suitable scheme is not trivial. There is no explicit and general understanding of the relationship between the context characteristics and the policies. Such understanding is essential to assist the choosing process and, consequently, adapt policies according to the context. More specifically, there are no overall directions or categorization in which context may influence policy behavior. Yet, regardless of the isolated evidence of individual works reporting their contexts and impacts on the policies’ performance, there is no comprehensive work discussing a unified view of the different contextual characteristics and their effects on the policies. The delimitation of context characteristics and their common effect can enhance and substantiate the caching management and the design of caching solutions.

Despite the contributions of previous literature reviews related to caching policies and ICN aspects (Ahlgren et al., 2012; Bari et al., 2012; Zhang, Li & Lin, 2013; Tyson et al., 2012; Xylomenos et al., 2013; Fang et al., 2014; Amadeo et al., 2014; Zhang, Luo & Zhang, 2015; Abdullahi, Arif & Hassan, 2015; Fang et al., 2015; Ioannou & Weber, 2016; Amadeo, Campolo & Molinaro, 2016; Saxena et al., 2016; Din et al., 2017), there is a lack of guidelines to understand context characteristics and their effect on the cache replacement policies in ICNs. Furthermore, surveys on web cache replacement policies (Wang, 1999; Podlipnig & Böszörmenyi, 2003; Balamash & Krunz, 2004; Panda, Patil & Raveendran, 2016) do not address this subject. To the best of our knowledge, there is no broad investigation on cache replacement schemes for the ICN domain or an integrated vision of the impacts of different context characteristics in the policy choice process. As a result, the lack of suitable schemes hinders the more efficient use of available cache resources, and therefore the effective extraction of the caching service expected benefits.

In this article, we present a systematic literature review (SLR) that, in contrast to previous works, investigates evidence in the literature about the effects of context aspects on cache replacement schemes’ performance. SLR is a straightforward and consistent process to compile evidence to answer a set of research questions and help further understand the evidence reported. To this end, we first cataloged the cache replacement schemes used in ICNs. The current literature presents various proposed strategies exploring different context aspects to enhance the eviction logic, aiming to achieve more potentially precise and customized techniques. We mapped context dimensions related to the content, network, node, and human aspects. We then categorized the respective context properties used by the replacement schemes proposed for ICNs. With the context properties, we provide a taxonomy of context dimensions and a policy categorization accordingly. Taxonomies may support the choosing process in the absence of the overall understanding of specific network contexts and what influences policy behavior.

In addition to the taxonomy, we compiled the context variations with reported relevant impacts on the policies, especially those leading to changes in the policies’ performance rank. This SLR was able to identify common context factors that differentiated the choice of best policy performance. Even so, as expected, there is no single optimal strategy to meet the requirements of all surveyed network contexts, since the performance of the caching policies varied according to the characteristics of each network. Last, we extended the SLR results with the analysis of proper context dimensions to be explored by the eviction process in different emergent networks, such as information-centric Internet of Things (Arshad et al., 2018; Dong & Wang, 2016), vehicular named-data networking (Khelifi et al., 2020), and in-network cache-based edge computing solutions (Zhou et al., 2017; Zhang et al., 2018). These emergent networks have gained attention from the research and industry communities, fostering the evolution of heterogeneous ICN solutions. The taxonomy and policy classification presented in this article can help to infer the choice among current or new policies adapted to these networks to ensure better network performance. Hence, the contribution of this article is threefold. It (i) provides a classification of contexts to assist those engaged in the design of adaptive caching solutions for ICN that target the more efficient use of available cache resources; (ii) substantiates the reasoning of the caching policy decision process during the design of caching systems by presenting and analyzing information from previous works; and (iii) contributes to the set of knowledge on caching systems regarding emergent networks and underpins context-aware caching solutions.

The remainder of the article is organized as follows. Background section presents the basic concepts of ICN and cache replacement schemes. The following section outlines the SLR methodology process, with the definition of the leading research questions. Results and analysis section presents the SLR results, with answers to the research questions and analysis of the main findings. In the ‘Applications’ section, this work contributes with a discussion of emergent networks and the association with context dimensions that can be explored to enhance the cache eviction process. The following section discusses relevant research directions. Finally, the last section concludes the article.

## Background

In this section, we present introductory concepts of ICN architectures and cache replacement policies.

### Information-centric networks

Information-centric networking is a new Internet architecture proposal widely discussed in the literature designed to meet the current de facto usage pattern of the Internet: the dissemination of content, such as videos and web pages. ICN comprises interconnected core functionalities for content naming, caching, and routing/forwarding to natively provide a content dissemination network. In its fundamental concept, the content name becomes an essential element for network routing, enabling the decoupling of content location from the content delivery process. Allied to that, ICN replicates contents in caches distributed across the network at the routers, and the closest copy will be returned when a user requests a content. Beyond the advantages of caching that provide reductions of network congestion, server loads, and access latency, the premise of independence of content location paves the way for efficient content distribution. Therefore, it adds advantages to ICN architectures, such as native support for mobility and multicast communication. The informational RFC 8763 (Rahman et al., 2020) presented by the Internet Research Task Force (IRTF) Information-Centric Networking Research Group (ICNRG) discusses some approaches for the real-world deployment of ICNs and trial experiments. Besides the clean-slate approach, there are directions for its coexistence with the TCP/IP—for example, the ICN adoption as an overlay network. The overlay approach proposes ICN islands deployed over existing IP infrastructure and connected using tunneling solutions. In this way, ICN packets are encapsulated inside IP packets through ICN/IP tunnels. Madureira et al. (2020) propose a resembling overlay approach with an SDN-based core network connecting edge networks operating NDN. In that case, the SDN core network encapsulates the NDN packet. Another approach is ICN as an underlay network, with the ICN islands connected to the Internet through proxies or protocol conversion gateways.

The literature presents several ICN architectures, such as Data-Oriented Network Architecture (DONA) (Koponen et al., 2007), Content Mediator architecture for content-aware Networks (COMET) (Garca et al., 2011), MobilityFirst (Raychaudhuri, Nagaraja & Venkataramani, 2012), and the previously mentioned NDN. They explore different architectural decisions about the naming scheme, caching, and routing processes (Xylomenos et al., 2013). Overall, the support for in-network caching is an essential feature of ICN design. In general, every router works with a CS structure to temporally store the contents. This way, when a router receives a content request, the router verifies whether the content is present in its own CS and immediately returns the content if stored locally. Otherwise, the router will forward the request to another destination.

**Figure 1:**
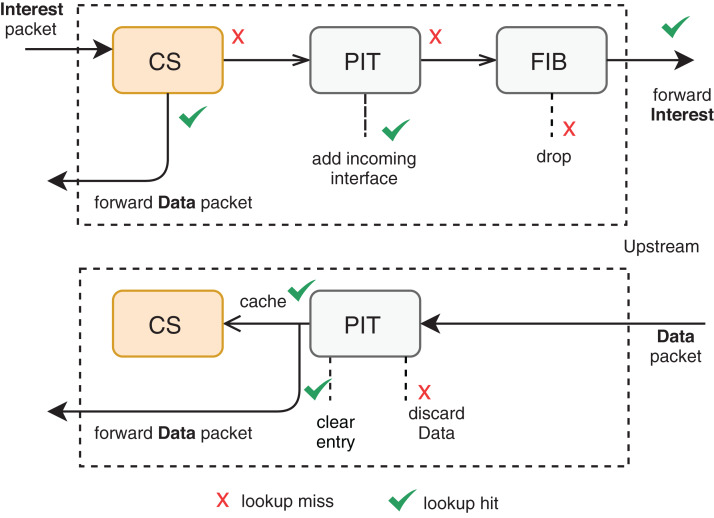
Packet forwarding engine at an NDN router (Zhang et al., 2014).

Among the different architectures, NDN outstands as a recent and promising trend to substitute (or coexist with) the current TCP/IP model. In NDN, each CR has three main structures to support in-network caching: CS, Pending Interest Table (PIT), and Forwarding Information Base (FIB). Figure 1 illustrates an overview of the interaction among these structures. A content request comes in the form of an *Interest* packet to the CR, which returns a copy of the content in a *Data* packet format if the content is already present in its CS for the same incoming interface of the Interest packet. Otherwise, a new PIT entry records a pending Interest with the respective incoming interface, and the CR forwards the Interest packet according to some named-based protocol. Multiple interests for the same data are aggregated in the same PIT entry. Once the Data-packet arrives at the CR, the corresponding PIT entry is satisfied by forwarding the data to the saved interfaces. The CS will, therefore, store the passing data according to some cache management protocols.

There are different policies to tackle the management of the CS structure. They can be classified as placement and replacement policies. Placement policies, also called insertion policies, target the decision of whether a passing content should be stored locally. Examples of placement policies include Leave Copy Everywhere (LCE), Probabilistic caching (Prob), Leave Copy Down (LCD) (Laoutaris, Syntila & Stavrakakis, 2004), Betweenness Centrality (Betw) (Chai et al., 2012), ProbCache (Psaras, Chai & Pavlou, 2012), and CRCache (Wang et al., 2014b). On the other hand, replacement policies are methods used to choose which content to evict from the cache when there is the need for storing new content, and no more space is available. Examples of replacement policies include LRU, LFU, Random, First-In-First-Out (FIFO), Recently/Frequently Used (LRFU) (Lee et al., 2001) and Recent Usage Frequency (RUF) (Kang, Lee & Ko, 2012). This work focuses on replacement policies, as we detail in the following sections.

### Cache replacement policies

Cache capacity tends to be a small segment of the amount of distinct content distributed over the network. Thus, it is essential to have an efficient eviction scheme among the cache management protocols. A replacement policy ensures that the content most expected to be accessed in a short time will remain in the cache, and the policy will, therefore, elect to evict the content that is less expected to be accessed. The performance gain of a network of caches like ICN depends on the reliability of the cache management, and different policies lead to different performance.

Traditional policies, such as LRU, LFU, or FIFO, are eviction strategies inherited from computer memory systems and are commonly used in ICN and web-proxy caching domains. These policies have been massively explored to analyze cache characteristics and the performance of complex network contexts through approximation models. Orthogonally, they were not designed to fit the needs of a network of caches and do not explore its potential. Thus, the literature presents a variety of newly proposed schemes. Jin et al. (2017) surveyed solutions for mobile caching in ICN, and among the contributions, they briefly described sets of cache insertion and replacement policies. Besides the usual LRU, LFU, FIFO, and simple Random, the list of replacement policies includes LRFU, LRU-k (O’neil, O’neil & Weikum, 1993), Time Aware Least Recent Used (TLRU) (Bilal & Kang, 2014), Aging Popularity-based Caching scheme (APC) (Li, Liu & Wu, 2013), Frequency-Based-FIFO (FB-FIFO) (Gomaa et al., 2013), and Adaptive Replacement Cache (ARC) (Megiddo & Modha, 2004). However, there is no broader study on replacement schemes for ICN domains. This SLR cataloged the schemes proposed for ICN to investigate contextual influences on the policies. Therefore, this work does not seek to discuss individual policies, and the reader can refer to the original literature to further information.

## Systematic literature review methodology

**Figure 2:**
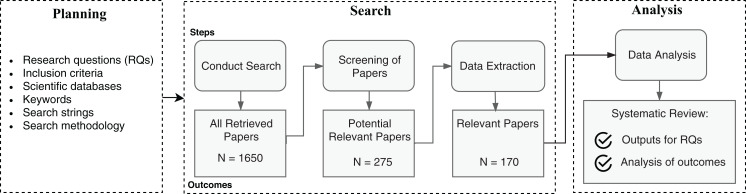
Steps of the SLR process.

The SLR methodology specifies a well-defined searching protocol, with the definition of research questions, research strings, explicit inclusion criteria of articles, among other steps. The methodology used in this article follows an adaptation of previously adopted SLRs in the Software Engineering discipline (Kitchenham & Charters, 2007; Petersen et al., 2008). Figure 2 summarizes the adopted SLR process.

The planning process ensures delimitation of the search scope with the definition of leading research questions, inclusion criteria, and the necessary inputs to operate the search. The search process is the article triage phase to collect relevant works and extract meaningful data that match the research questions. The data analysis evaluates the extracted data to summarize the primary evidence and point contributions. The following subsections detail the planning, searching, and analysis processes.

### Planning process

This study aims to map context information associated with the performance of cache replacement strategies to help the choosing and design process while applying ICN. Since the scope and definition of context information can be relative to the research domain, we intended to characterize relevant dimensions surrounding the cache replacement schemes. Additionally, we also intended to identify the cache replacement strategies applied and their context characteristics, and investigate reported evidence about how the identified context information influences the behavior of cache replacement policies in ICNs. To this end, we defined the following research questions (RQs):

RQ1: What is context from the perspective of a cache replacement policy?
RQ2: Which are the context characteristics used by the policies?
RQ3: Which are the cache replacement strategies applied for in-networking caching in ICN?
RQ4: What context variations had effects on the performance of the cache replacement strategies?

Notice that the research questions correlate with each other in the sense that they rely on each other’s outputs in different ways: the first three questions are requisites to answer the last question; to answer RQ2 it is necessary a primary overview direction for RQ1 and also the output for RQ3; the complete delimitation of context that answers RQ1 is an iterative process that relies on RQ2 and RQ3 outcomes.

After the definition of the research questions, we specified a list of relevant keywords based on the analysis of manually selected articles, and we defined correspondent search strings using AND and OR operators, as shown in Table 1. The search strings were meant to drive automatic searches on relevant research engines. We adapted the search strings according to the syntax of the scientific databases.

**Table 1:** Search string example.

(“ICN” OR “NDN” OR “CCN” OR “information centric” OR “information-centric” OR “named data” OR “named-data” OR “content centric” OR “content-centric”) AND (“cache” OR “caching”) AND (“replacement” OR “eviction” OR “performance” OR “management” OR “policy” OR “policies”)

The selection criteria included works written in English, addressing any aspects of cache replacement policy comparisons in ICNs. We also had the articles approaching new schemes for the eviction process for ICNs as part of the contributions.

### Searching process

The first step of the searching process was applying the automatic searches as specified in the planning phase. We did not set a lower year threshold in the search databases for the publication year range, and the upper bound was set to 2019. We cataloged a total of 1,650 articles in this phase. In the following, the screening process comprehended abstract reading and analysis of all matched articles, to filter according to the inclusion criteria. Upon abstract filtering, we obtained 275 articles. Those were potential works where we could find answers to the predefined research questions. Finally, we performed full article reading and analysis of the potential works to extract relevant information and evidence about the research questions. As a result, we reached a total of 168 articles pertinent to our research. Additionally, we incremented the results by carrying out a non-systematic snowballing research process on the read articles and search engines to update with new works not covered in the first search. This process resulted in the addition of two relevant documents.

### Analysis process

The resulting articles and their correspondent extracted data consisted the input for our study. In this phase, we categorized and correlated data from different articles to empirically mining relevant information patterns. We report our main findings regarding the research questions in the following section.

## Results and analysis

The SLR process described in the previous section enabled us to answer the main research questions introduced in this manuscript. The following subsections describe the process to accomplished this.

### RQ1: context dimensions

Many definitions of context have been given in the literature as well as different methods to model and design context-aware applications (Abowd et al., 1999; Bettini et al., 2010; Dey, 2001; Liu, Li & Huang, 2011; Vieira, Tedesco & Salgado, 2011; Alegre, Augusto & Clark, 2016; Van Engelenburg, Janssen & Klievink, 2019). Although there is no single consensual definition, they all converge on the importance and benefits of integrating the awareness of any relevant information from relevant entities with the computational environment.

As a result of the literature review analysis process, our definition of *context* comprises, in a broad sense, information that can be used by the policy as input data to direct the eviction process. Also, it includes information “external” to the policy that can be used within a computational environment and could influence the policy’s performance.

To understand and delimit what entities could represent the context from the perspective of cache replacement strategies, we direct the article reading and extraction of possibly relevant information based on leading questions related to the content. Since the process of dealing with contents is the overall purpose of having caching policies, we placed content as a feedstock for caching policies, and we defined questions from the content’s point of view, as follows:

- *What content is being requested?* In this dimension, we seek for characteristics of the content itself (and the application), such as content size, popularity, type;
- *When is the content requested?* This dimension specifies time-related information regarding the content and its relation to the user—for instance, time of access, time of creation, or user delay to receive the content.
- *Where is the content located and distributed?* This dimension specifies network characteristics, such as topology and link capacity, and features about the node/routers that store the content, such as cache capacity and the number of interfaces.
- *Who is requesting the content? Also, who is publishing the content?* This dimension relates to the human aspect, in which preferences, behavior, and routines are mapped as a context dimension. The dimension can also refer to machine-to-machine communication, but, in this case, the characteristics overlap with information of the node contemplated in the previous dimension.

**Figure 3:**
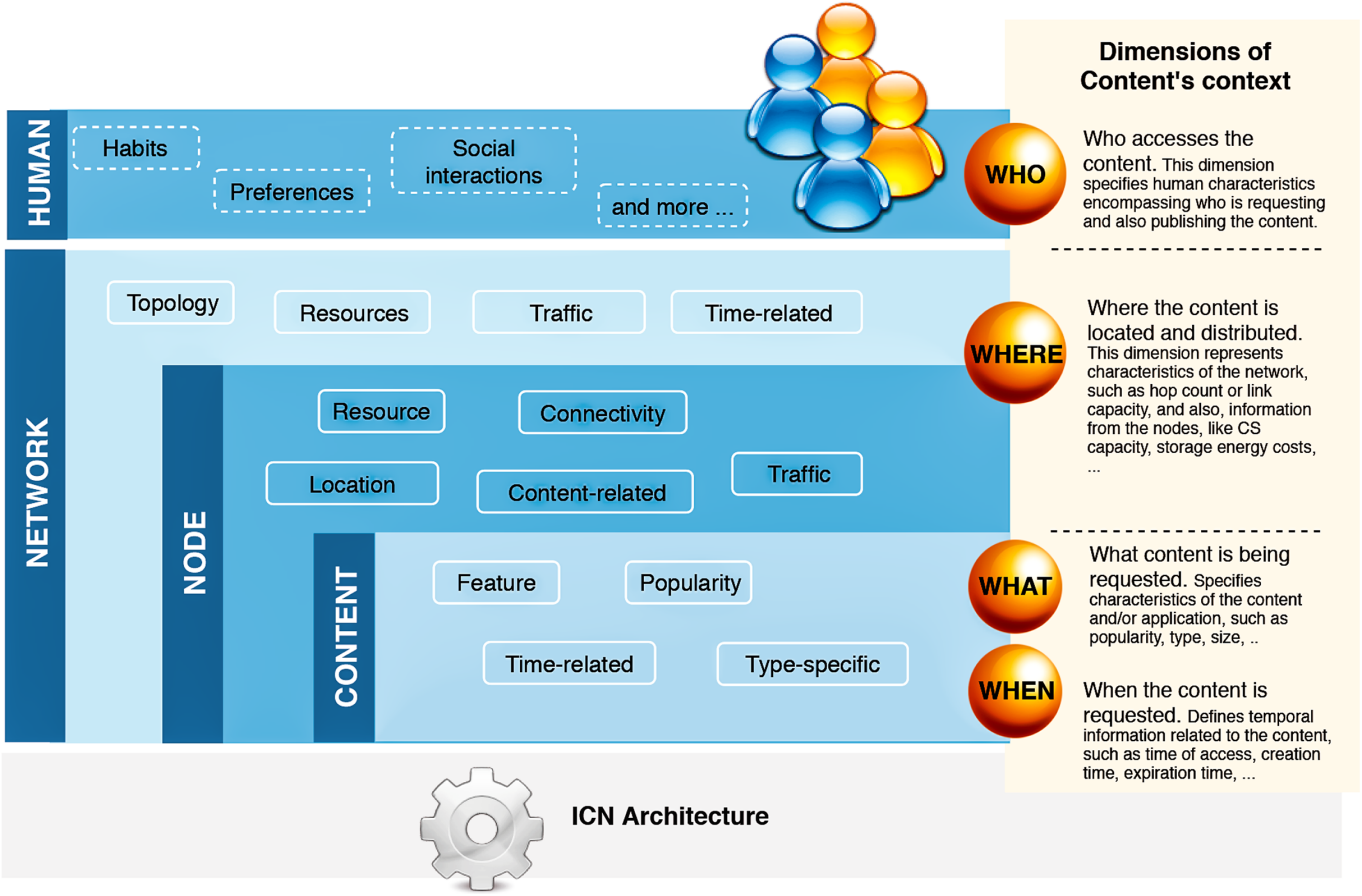
The hierarchy of context dimensions identified from the surveyed articles and the proposed classification for the correspondent characteristics associated with the cache replacement schemes.

Therefore, we extracted relevant information that would apply to these dimensions and correlate with the cache replacement schemes. Based on the extracted data, we characterized context dimensions according to four main categories: *network, node, content*, and *human*. Figure 3 illustrates the hierarchy of our classification. A *context view* is represented by current information of cached content in a particular node, which belongs to a network, and is accessed or produced by a user. Each of these dimensions contains properties related to the cache eviction process in one or more of the surveyed articles. We detail the list of properties in the next subsection.

Additionally, we also consider ICN architecture decisions as part of the context. The other cache-related protocols, such as placement policies or naming schemes, are relevant aspects and should be included as part of the context. This article surveyed the impacts of different architecture decisions on the replacement schemes; however, the discussion of specific caching protocols properties is out of the scope of this work.

### RQ2: context characteristics

**Figure 4:**
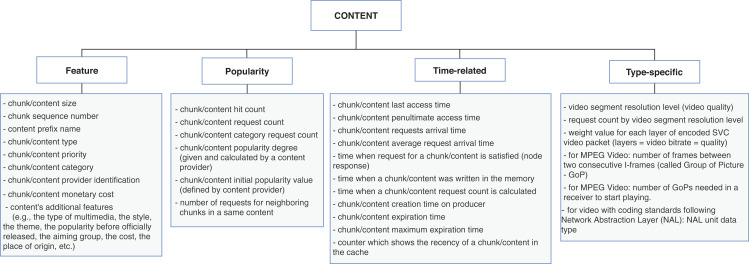
Properties from content dimension extracted from the cache replacement schemes for ICNs.

**Figure 5:**
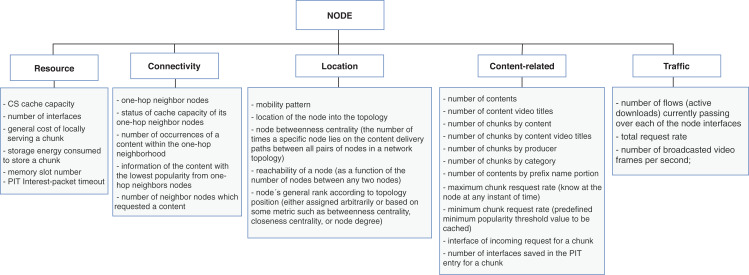
Properties from node dimension extracted from the cache replacement schemes for ICNs.

**Figure 6:**
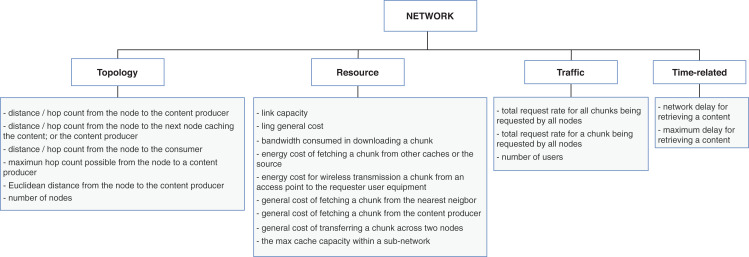
Properties from network dimension extracted from the cache replacement schemes for ICNs.

Our second research question aims at identifying the context characteristics directly related to the policies. To this end, we collected the types of information used as input data for the replacement schemes and classified correspondent properties for the main context dimensions of Fig. 3. We further discuss the context characteristics as follows:

- The **content** dimension is subcategorized into four types of properties: *feature, popularity, time-related*, and *type-specific*. The *feature* properties are global ones, that is, are inherent to the content and usually do not vary according to the other context dimensions. Conversely, *popularity* and *time-related* properties are related to the node caching the content, and consequently, their values differ from node to node. The *type-specific* subcategory is reserved for specifying singular aspects of data or application types. Figure 4 contains a list of properties extracted from the surveyed articles for the content dimension. In this case, the type-specific properties are mainly about video content, for illustrative purposes.
- The **node** dimension is subcategorized in *resource, connectivity, location, content-related*, and *traffic*. Accordingly, the *resource* properties are inherent to the node; *connectivity* and *location* features are mostly related to neighbor nodes and the position of the node into the topology. The *content-related* represents the intersection with the content’s dimension and gathers content’s information in a broader granularity. The *traffic* properties are related to the flows of data traffic passing through the node. Figure 5 shows the list of properties extracted from the surveyed articles for the node dimension.
- The **network** dimension represents properties common to general network types. The properties are categorized into four classes: *resource, topology, traffic*, and *time-related*. The *resource* class groups the overall network capabilities, such as bandwidth, link capacity, and fetching content costs. The *topology* properties are more specific about network’s size, represented mainly with the distances between nodes. The *traffic* class has the same connotation as in the node dimension but differs in granularity, and the *time-related* class defines temporal properties. The presented properties in the time-related class are similar to some of the topology properties. They are related to the distance between nodes measured in time units to reflect the delay to retrieve content. Figure 6 presents the list of properties extracted from the surveyed articles for the network dimension.

The previous list of properties is a broad definition of context characteristics to assist in the analysis of cache replacement schemes. It helps to visualize what dimensions are directly related to the policies and could significantly impact the applied network context. However, it is not a static list and can be increased as new information becomes available and relevant to a specific ICN instance. Furthermore, some of those properties are closely related to more than one context dimension. It is possible to change their perspective in terms of classification to represent a given ICN context. Moreover, the unified view of properties can substantiate the design of novel cache solutions by helping to identify potential gaps for new situations.

The **human** dimension is an emergent and new approach to be explored as part of the context. Recent research fields like *people-centric networking* (Conti et al., 2015) and *human-centric multimedia networking* (Rosário et al., 2016) are gathering attention to the basic fact that users play an essential role in demanding contents or network services, and different human characteristics can lead to different impacts on the network. In this way, human attributes are potential drivers in the design of network solutions. The many examples of human data, such as *behaviors, interests, personality, character, social interactions, humor, daily routines, gender, age, etc*., opens up a range of possibilities to be explored. Pires et al. (2018) performed experiments with real user data and associated distinct user habits with different cache replacement policies. The work reinforces the relevance of the human dimension for network configuration. However, it is an incipient research field, and there is still a lack of studies intersecting human features with caching policies. Thus, it was unsuitable for proposing a proper classification of properties for the human dimension in the current research. Moreover, although some policies intended to incorporate features related to the user in the caching process (Al-Turjman, Al-Fagih & Hassanein, 2013; Xing et al., 2017; Zhang, Tan & Li, 2018), the human characteristics are not directly used by the policies. For instance, Wei et al. (2014) proposed a mobility-aware caching strategy for mobile networks in which they model the transition of users among WiFi access points as a stationary Markov model. In a broad sense, the user’s mobility has the same connotation as the node’s mobility. In the surveyed works that deal with mobility, the concept of a node’s mobility suits the objectives since the human dimension is not directly associated with mobility patterns. Different user’s profiles can be associated with different mobility patterns, for example, different ages or professions (Liang et al., 2012) or even different personalities (Chorley et al., 2013).

### RQ3: cache replacement schemes for ICNs

The literature shows various proposed replacement schemes for ICNs exploring beyond the context of the content and adding properties of node and network’s dimensions. In this direction, we cataloged the replacement schemes applied to the surveyed articles to collect context features and understand their correlations. To better readability, we classified the schemes according to the classes of context information they used. They are classified in: *content-based; content and network-based; content and node-based; content, network and node-based;* and *network and/or node-based* schemes. Tables 2–6 contain the lists of the cache replacement schemes in each class, respectively. The tables also detail the correspondent context property categories used by the policies, which reveal the diversity of context combinations explored in the literature. We grouped the policies accordingly. This classification provides a comprehensive view of what context information the techniques required. Therefore, it is the first guide to map which context variances could directly influence the performance of the technique.

**Table 2:** Content-based cache replacement schemes.

Content property categories	Replacement schemes
Popularity	Rossi & Rossini (2011), Chao et al. (2013), Ran et al. (2013), Yeh et al. (2015), Nakayama, Ata & Oka (2015), Liu, Zhu & Ma (2016), Zhao et al. (2017), Kalghoum & Gammar (2017), Sinky et al. (2018), Li, Yu & Li (2018), Kalghoum & Saidane (2019)
Time-related	Ravi, Ramanathan & Sivalingam (2014), Li, Ma & Hu (2015a), Rezazad & Tay (2015), Rhaiem, Fourati & Ajib (2016), Shukla & Abouzeid (2017), Dhiab et al. (2017), Vural et al. (2017), Hou et al. (2019), Meddeb et al. (2019), Din et al. (2019)
Popularity and time-related	Wang et al. (2012a), Neves dos Santos et al. (2013), Qian et al. (2014), Chen et al. (2014), Abidi & Gammar (2015), Xin et al. (2016), Yao et al. (2018), Chootong & Thaenthong (2017), Zhang, Tan & Li (2018), Huang et al. (2018), Tang et al. (2019)
Popularity, time-related and feature	Kang, Lee & Ko (2012), Bilal & Kang (2014), Han et al. (2014), Bilal & Kang (2017), Sri Prakash & Moharir (2018), Sertbaş et al. (2018)
Time-related and feature	Thomas & Xylomenos (2014), Rao, Schelen & Lindgren (2016), Wu et al. (2014), Tarnoi et al. (2019)
Popularity and feature	Chandrasekaran, Wang & Tafazolli (2015), Chandrasekaran et al. (2018), Lee & Hong (2017)
Popularity and type-specific	Jia et al. (2016), Ge et al. (2016)
Time-related and type-specific	Zhang et al. (2017c)
Time-related, type-specific and feature	Ghahfarokhi, Moghim & Eftekhari (2017)
Type-specific and feature	Lee, Lim & Yoo (2013)
Popularity, time-related, type-specific and feature	Lee, Lim & Yoo (2013)

**Table 3:** Content and Node-based cache replacement schemes.

Content property categories	Node property categories	Replacement schemes
Popularity	Location	Wei et al. (2014), Chen et al. (2016), Mick, Tourani & Misra (2016), Lal & Kumar (2019)
	Content-related	Lal & Kumar (2016), Zhang, Tan & Li (2017b), Baugh & Guo (2018)
	Traffic	Saltarin et al. (2018)
	Traffic and connectivity	Yang & Choi (2018)
	Traffic and location	Liu et al. (2019b)
Popularity and time-related	Traffic	Karami & Guerrero-Zapata (2015), Rocha et al. (2016), Zhou & Ye (2017), Khan & Khan (2017), Qu et al. (2018)
	Connectivity	An & Luo (2018)
	Content-related and traffic	Yao et al. (2019)
Time-related and feature	Content-related	Hahm et al. (2016)
	Connectivity and location	Aoki & Shigeyasu (2017)
Popularity, time-related and feature	Connectivity	Wood et al. (2013)
	Content-related and traffic	Ong et al. (2014)
Popularity and feature	Content-related	Li et al. (2015b), Dron et al. (2013)

**Table 4:** Content and Network-based cache replacement schemes.

Content property categories	Network property categories	Replacement schemes
Popularity	Topology	Wang et al. (2011), Wang, Bi & Wu (2012b), Ming, Xu & Wang (2012), Ren et al. (2014), Hu et al. (2015), Huang et al. (2017), Khan et al. (2018)
	Resource	Caarls, Hargreaves & Menasché (2015)
	Traffic and time-related	Sinky et al. (2018)
Popularity and time-related	Topology	Chen, Fan & Yin (2013), Ostrovskaya et al. (2018)
	Time-related	Yokota et al. (2016)
	Resource	Pal & Kant (2017)
Popularity and feature	Resource	Wang, Bayhan & Kangasharju (2015)
	Time-related	Sun & Wang (2015)
	Resource and time-related	Ndikumana et al. (2018)
Popularity, time-related and feature	Topology	Duan et al. (2013)
Time-related	Time-related	Dai et al. (2017)
Feature	Resource	Xing et al. (2017)

**Table 5:** Content, node, and Network-based cache replacement schemes.

Content property categories	Node property categories	Network property categories	Replacement schemes
Popularity and feature	Content-related and location	Topology	Panigrahi et al. (2014)
	Content-related and traffic	Traffic	Liu et al. (2018)
	Traffic	Resource	Badov et al. (2014)
	Resource	Time-related and resource	Gür (2015)
Popularity and time-related	Content-related	Topology	Rath, Panigrahi & Simha (2016)
	Traffic and location	Topology and time-related	Al-Turjman, Al-Fagih & Hassanein (2013)
Popularity	Traffic	Topology	Chen et al. (2017)
	Connectivity	Topology and resource	Zhang et al. (2016)
Time-related	Resource	Topology and resource	Llorca et al. (2015)
	Location	Topology	Naz, Rais & Qayyum (2016)
Popularity, time-related and feature	Traffic	Topology and time-related	Al-Turjman (2017)

**Table 6:** Node and/or network-based cache replacement schemes.

Node property categories	Network property categories	Replacement schemes
Content-related and location	Topology	Wang & Bensaou (2012a, 2012b), Yanuar & Manaf (2017)
Content-related and resource	Time-related	Sureshjani & Moghim (2018)
Resource	Resource	Wang et al. (2014a)
–	Resource	Ioannidis & Yeh (2016, 2018)

**Figure 7:**
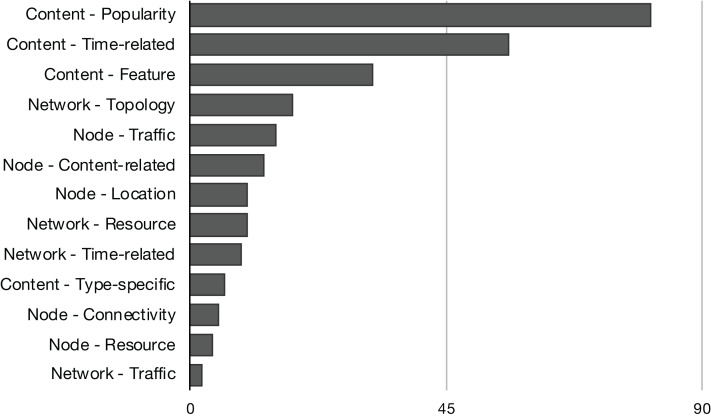
Distribution of context properties categories according to the number of policies that used the correspondent properties in their eviction logic.

The *content-based* replacement policies explore only the characteristics of the content to make the eviction decision. They use one or more of the content features listed in Fig. 4. Following this reasoning, the policies that explore beyond the content and start to look to characteristics of the node or the network that could lead the eviction process to make a better decision are classified accordingly. They also use one or more features listed in Fig. 5 or Fig. 6. Naturally, almost all the schemes further explore the content dimension; however, we also found methods dealing only with network and node features to assist the eviction process. Figure 7 illustrates the usage distribution of context properties by their categories. We ranked the context categories according to the number of policies that used one or more of the corresponding category properties. After content popularity, time-related and feature properties, the network topology and node traffic properties are the most used ones. It is important to remark that for the classification of policies, we did not account for the general use of node CS cache capacity and the number of interface information, since it can usually be part of the caching process.

### RQ4: effects of context variation

Our objective in this section is to carry out an evidence-based analysis and identify what context dimensions can affect the policies’ performance. An evidence-based analysis can increment and drive approximate solutions to the problem of finding the optimal policy. The choice of a best-fitting replacement policy exponentially grows in complexity when there is a diversity of context variables. Many efforts have been employed to comparatively evaluate different policies in different network scenarios. Usually, the evaluations comprises variations of context like cache size, topology, or content popularity. The results gives us approximations and insights about which policy performs better in the evaluated scenarios or which variations in context can impact the policy’s decisions. Such information is essential to help the process of network design when deciding which replacement policy should be instantiated in a given network scenario. In this way, we collected *reported evidence* from the surveyed articles about the effects of context variations on replacement schemes’ performance.

We have found policy comparisons in different scenarios with variations of many aspects like request rates, forwarding strategies, number of consumers, number of contents, and overall topology. Nevertheless, in summary, we found that variations in the *node location, cache size, cache placement policy* and *content popularity* had some relevant effect on the policies’ performance. The first three presented variations resulting in different choices of replacement policies. Also, beyond the impact on the choosing point of which cache replacement schemes to apply, variations in *cache size* and *content popularity* presented other relevant effects related to the policies’ performance. We discuss the context variations separately in the following. To support the reading, Table 7 presents a description of the policies reported in this section.

**Table 7:** Set of content placement and replacement policies.

Abbrev.	Policy name	Type	Description	References
LRU	Least recently used	Replacement	Removes the last accessed content in the cache	–
LFU	Least frequently used	Replacement	Removes the last frequently used content in the cache	–
FIFO	First-in-first-out	Replacement	Removes the oldest content placed in the cache	–
–	Random	Replacement	Removes one content randomly	–
–	Size	Replacement	Removes the content with largest size in the cache	Abrams et al. (1996)
LRFU	Least recently/frequently used	Replacement	Considers the recency and frequency of contents to compute a Combined Recency and Frequency (CRF) metric. CRF values are higher for more recent and frequent contents. The policy evicts contents with lower CRFs	Lee et al. (2001)
FCDC	Fast convergence caching replacement algorithm based on dynamic classification method	Replacement	Considers categories of contents by content’s popularity and a popularity rank by categories. Contents in lower ranked categories can be evicted for ones in higher ranked categories	Chao et al. (2013)
RUF	Recent usage frequency	Replacement	Considers categories of contents by similarity and a popularity rank by categories. Contents in lower ranked categories can be evicted for ones in higher ranked categories	Kang, Lee & Ko (2012)
EV	Energy efficiency cache scheme based on virtual round trip time	Placement/replacement	Considers the energy consumption to store and to transport the content. Places the contents with storage energy smaller than their transport energy, and compares the energy saving of the cached contents with the energy saving of the passing content to evict the contents	Wang et al. (2014a)
PBRS	Content-popularity and betweenness based replacement scheme	Replacement	Removes the content with the lower popularity. Computes the content popularity based on the content’s requests and node’s betweenness centrality	Liu et al. (2019b)
ABC	Age-based cooperation	Replacement	Removes the content based on content’s Time-to-Live (TTL). Computes TTL based on the node’s location in the topology and the content popularity. The closer to the edge and/or the more popular a content, the longer its TTL value. Also called TTL	Ming, Xu & Wang (2012)
2Q	Two queues	Replacement	Designed for buffer management, it considers two lists of pages. The first list applies FIFO in the incoming page requests. The second list receives the pages in the first list requested again and their subsequent requests and applies LRU	Johnson & Shasha (1994)
ARC	Adaptive replacement cache	Replacement	Designed for buffer management, it considers two LRU lists. The first list contains pages requested once in a recent time, and the second list pages requested at least twice. The policy adaptively decides the number of pages to maintain in each list according to the workload characteristic	Megiddo & Modha (2003)
LIRS	Low inter-reference recency set	Replacement	Designed for buffer management, it considers the number of other pages accessed between the last and penultimate access for a page as Inter-Reference Recency (IRR) metric. The policy removes the page with the largest IRR	Jiang & Zhang (2002)
MQ	Multi-queue	Replacement	Designed for buffer management, it considers multiple lists with different access frequencies for different periods	Zhou, Philbin & Li (2001)
PPC	Popularity prediction caching	Replacement	Designed for video content. Predicts and caches the future most popular videos’ chunks based on the number of requests for neighboring chunks in the same video content. Evicts chunks with the least future popularity	Zhang, Tan & Li (2018)
CCP	Cache policy based on content popularity	Replacement	Considers previous content popularity and the number of hits in a current interval of time to compute the current content popularity. The policy evicts less popular content	Ran et al. (2013)
Betw	Betweenness centrality	Placement	Considers the node’s position at the topology in terms of node’s centrality measures to place the content. Only selected nodes with higher measures cache the content. Also called Leave-Copy-Betw (LCB), or Centrality	Chai et al. (2012)
LCD	Leave copy down	Placement	Places the content only in the immediate downstream node of a cache-hit point	Laoutaris, Syntila & Stavrakakis (2004)
LCE	Leave copy everywhere	Placement	Places the contents in all caches along the reverse path of the content request	Laoutaris, Syntila & Stavrakakis (2004)
Prob	Probabilistic caching	Placement	Each cache in the reverse path of the content request stores the content with a constant probability *p*. Also called Leave-Copy-Probabilistically (LCP)	Laoutaris, Syntila & Stavrakakis (2004)
–	ProbCache	Placement	Considers the shared storage capacity of the request path and the node’s distance to the content producer to calculate the node’s probability of caching the content; Also called PProb	Psaras, Chai & Pavlou (2012)
–	CRCache	Placement	Considers the content popularity and the node’s centrality measures to calculate the probability of caching the content. The most popular contents are cached in the nodes with the highest centrality. Also called Cross	Wang et al. (2014b)
PCP	Progressive caching policy	Placement	Considers the immediate downstream node of a cache-hit point to store the content, the number of interfaces saved in PIT entry for the intermediate nodes, and the number of requests for edge nodes	Wang & Bensaou (2012a)
Rand	Single node random caching	Placement	Places the contents in one random intermediate node along the delivery path	Eum et al. (2012)

#### Node: location

The works from Wang & Bensaou (2012a), Tarnoi et al. (2014), Gallo et al. (2014), Li, Simon & Gravey (2012) and Newberry & Zhang (2019) presented evidence of the impact of node’s location on cache replacement scheme choice. Table 8 summarizes the characteristics of the scenarios that supported the analyses. In the following, we discuss the reported impacts:

- Wang & Bensaou (2012a) proposed two complementary replacement algorithms to handle different workload characteristics observed by both edge and intermediate router nodes. The eviction logic uses the hop count factor to prioritize the maintenance of more distant contents and, consequently, reduce network resource consumption. Besides the hop count, the replacement algorithm for intermediate nodes considers the number of node’s interfaces saved in the PIT entry for a content to estimate the diversity of the content requests. The proposed solution outperforms homogeneous configuration with LCE + LRU, and the results emphasize the benefits of using heterogeneous replacement policies according to the location of the node into the topology. However, the eviction solutions were evaluated only in conjunction with a proposed placement policy named PPC, limiting the analysis of the heterogeneous eviction solution separately. The proposed replacement schemes logic would be able to work together with other location policies, like LCE.
- Li, Simon & Gravey (2012) used the LRFU policy with a weighting parameter *y* to represent a multi-policy caching where every content router implements its caching policy according to its location in the network. The LRFU behavior can switch to be more closely similar to LRU or LFU according to the value of *y*. The router location is relative to his position between users and servers. The routers (CRs) are classified according to a defined “entering degree”, which represents the number of the shortest path connecting front-end CRs with servers via a CR. The reasoning to configure different values of LRFU parameter *y* comes from an experiment under an emulated European Backbone Ebone topology with 40 nodes, in which they performed experiments with homogeneous configurations of *y* in all routers. They observed that the routers with lower hit rate achieved their best performance with higher values of *y*, and on the contrary, routers with higher hit rates achieved their best performance with lower values of *y*. Allied to that, they also observed that the position of the router in the hit rate rank is directly proportional to his position in the topology, in the sense that the closer to the edge, the higher is the hit ratio performance.
- The experiments of Tarnoi et al. (2014) reveal the difference of performances between LRU and Random according to the node position. For the experiment with a cascade network scenario and one content requester, LRU and Random, in combination with LCE placement policy, interchange positions on the rank of the cache hit performance: for the level 1, LRU outperforms Random, but from level 2 onward, LRU performance decreases drastically and Random also slightly decreases but now with better performance than LRU. The difference in the rank of cache hit rate is similar for the scenario variation with multiple content requests, but LRU and Random interchange position after the third level node. For the Internet topology, the result groups edge and core nodes, and again, LRU presented the best results for edge nodes while Random for core nodes.
- Continuing the discussion about LRU and Random replacement policies, Gallo et al. (2014) came to a similar conclusion in terms of the difference in performance when varying node locations. For that, the authors presented an analysis of cache miss probability depending on the content popularity distribution. The analysis suggest that LRU and Random have significantly different performances only for popularity distributions highly concentrated on a relatively small number of objects. That difference is also relative to the position of the node in the topology. The more popular objects are more likely to be found at the edge node when using LRU, but those more popular objects can be more evenly distributed when using Random across the path. Also, the evaluation presents heterogeneous configuration for the leaves and root levels of a tree topology: LRU-Random and Random-LRU, also LRU-LRU and Random-Random. The heterogeneous LRU-Random configuration achieved better performance than the other configuration options, that is, LRU and Random configured respectively in the edge and intermediate levels.
- While evaluating the advantages of integrating big data applications in an ICN-like architecture, Newberry & Zhang (2019) argue the benefits of using different cache replacement policies at each layer of a data center fat-tree topology. They compared the performance of homogeneous and heterogeneous policy configurations, placing the cache in each node of a fat-tree topology with three layers, composed of 16 core, 32 aggregation, and 32 edge switches. They performed combinations of the policies LRU, 2Q, ARC, LIRS, and MQ, on the levels of the tree topology, totaling 125 combinations for each variation of cache size. The results could reveal the different behaviors at different layers of the topology and the suitability of different policies at each level. However, the gain of the reported best heterogeneous configurations concerning the best homogeneous configuration is not explicit in the article.

**Table 8:** Scenarios concerning replacement policies evaluations with different effects on the policy choice.

	Network			Node		Content		ICN architecture			
References	Topology	N. Cons./N. Prod.	N. Objects and Requests	Location of the node	Cache size	Popularity (Zipf(a))	Chunk size	Placement policy	Eviction policy	Metrics	Effect
Wang & Bensaou (2012a)	Internet-like; 32 CRs	–/2	80.000 objects, each with [1-2447] or [1-9548] chunks; Total of 300.000 object requests	Edge and Intermediate	[100–8,000] chunks	[0.92; 0.78] and [0.96; 0.74]	10 KB	LCE and PCP	LRU; Proposed edge and core	Hit rate; Hit gain; Path stretch	[*Node-location*] Different policies for different node locations; Limited evaluation
Li, Simon & Gravey (2012)	Internet-like; 40 CRs	1,000/3	500 objects, each with [60–120] chunks; Total of 45.000 chunks; Simulation time of 10.000 min	Edge and Intermediate	100 chunks	1.0	–	LCE	LRU, LFU and LRFU multi-γ	Hit rate; Number of access to server	[*Node-location*] Different configurations of LRFU for different node locations
Tarnoi et al. (2014)	Cascade; 5 CRs	1/1 and 4/1	1.000 objects; Simulation time of 10.000 s	Edge and Intermediate	10, 20, 50, and 100 objects	[0.4–1.6]	–	LCE and Prob	LRU, LFU and Random	Hit rate; Server load; Round trip hop distance	[*Node-location*] LRU and Random interchange positions for different node locations; [*Placement-policy*] Different eviction policies for different placement policies
Tarnoi et al. (2014)	Internet-like; 50 CRs	42/8	8.000 objects; Simulation time of 10.000 s	Edge and Intermediate	80, 160, 400, and 800 objects	[0.4–1.6]	–	LCE and Prob	LRU, LFU and Random	Hit rate; Server load; Round trip hop distance	*[Node-location]* LRU and Random interchange positions for different node locations. [*Placement-policy*] Different eviction policies for different placement policies
Gallo et al. (2014)	Tree; 3 CRs	2/1	20.000 objects	Edge and Root	[10–100] objects	1.7	–	LCE	LRU and Random	Miss probability	*[Node-location]* LRU and Random interchange positions for different node locations
Newberry & Zhang (2019)	Data Center Fat-tree; 80 CRs	–/128	–	Core, aggregation, edge	64, 128, 256, 512 and 1024 MB	–	128 KB	LCE	LRU, 2Q, ARC, LIRS and MQ	Total network traffic	[*Node-location*] [*Cache-size*] Different policies for different node locations, for different applications, and for different cache sizes
Chao et al. (2013)	–	–	500 objects; 50 requests/s; Simulation time of 1.080 s	–	[5–75] objects	1.0	–	–	FCDC, LRU and RUF	Hit rate	[*Cache-size*] LRU and FCDC interchange positions for different cache sizes
Wang et al. (2014a)	Cascade; 5 CRs	–	50 objects	–	aprox. [5–40] objects	0.8, 1.5, and 2.0	–	LCE and EV Placement	LRU, EV Replacement, and Popu	Energy efficiency	[*Cache-size*] Different combinations of placement and replacement policies for different cache sizes
Liu et al. (2019b)	Tree; 7 CRs	4 /1	1.000 objects; aprox. 100 requests/s; Simulation time of 200 s	Edge and Intermediate	[10–90] MB	0.7	–	LCE	PBRS, LRU, LFU and FIFO	Hit rate	[*Cache-size*] LFU and PBRS interchange positions for different cache sizes
Sun et al. (2014)	Internet-like; 80 K CRs	16 million/221	More than 500 K objects, with total volume of 137 TB; 196 million requests	Edge, Middle, and Core	1 GB, 10 GB, 100 GB, and 1 TB	1.174	–	LCE, LCD, Rand, Prob, PProb, Centrality and Cross	LRU, LFU, FIFO, TTL and Size	Hit rate; Traffic reduction; Server load reduction	[*Cache-size*] [*Placement-policy*] Different eviction policies for different cache sizes, and for different placement policies
Chen et al. (2016)	Wireless Mesh; 15 CRs	–	Total of 300 requests; Simulation time of 1 h	–	210 bytes	0.8	14 bytes	LCE, LCP, LCD, and LCB	LRU, LFU, Random, and FIFO	Hit ratio; Energy consumption	[*Placement-policy*] Different eviction policies for different placement policies

**Note:**

CR, Content Router; N. Cons./N. Prod., Number of content consumers/Number of content producers.

All the scenarios discussed in this subsection concluded that heterogeneous policy configurations achieved the highest performances than the homogeneous configurations. Whether for small topologies (Tarnoi et al., 2014; Gallo et al., 2014) or larger topologies (Wang & Bensaou, 2012a; Li, Simon & Gravey, 2012; Tarnoi et al., 2014; Newberry & Zhang, 2019), the works observed different traffic characteristics in the different nodes. They attributed this difference to the node position and associated different policies to different traffic profiles.

Multiple levels of caches naturally present that difference in traffic characteristics by cache-level due to the knowing *filtering-effect*. The filtering-effect happens any time a lower-level cache hits a content request. The cache does not propagate that request to the rest of the network and propagates only the miss requests to upper-level caches. This behavior modifies the original characteristics of the traffic. Many studies have been addressing the progressive filtering effect in hierarchical web caches (Williamson, 2002; Zhou et al., 2013; Melazzi et al., 2014). That filtering has a direct impact on the *temporal locality* of the requests (Jin & Bestavros, 1999). Temporal locality refers to the property that recently accessed objects are likely to be reaccessed in the near future. As cache levels filter requests, the temporal locality intensity becomes gradually weakening, and the traffic profile at upper-level caches becomes more random (Jin & Bestavros, 1999). That explains why Random policy achieved better performances for intermediate nodes in some of the discussed scenarios. As expected, workloads with temporal locality property have a strong correlation with caching policies (Garetto, Leonardi & Martina, 2016), and variations in the temporal locality patterns directly impact the variations of caching policies performances.

Regarding the context attributes explored by the replacement schemes, only two of the works presented evaluations including context features in the eviction logic that helped differentiate the node’s position: like the *node’s number of interfaces* (Wang & Bensaou, 2012a) and the *node degree* as a general rank according to the topology (Li, Simon & Gravey, 2012). However, other works are exploring those, and other context attributes that could be helpful. The context attributes with their respective classification and reference works are:

- Node-Location: node betweenness centrality (Chen et al., 2016; Liu et al., 2019b);
- Node-Location: reachability of a node (Panigrahi et al., 2014);
- Node-Location: node’s general rank according to topology position (Mick, Tourani & Misra, 2016; Aoki & Shigeyasu, 2017; Naz, Rais & Qayyum, 2016);
- Node-Content-related: number of interfaces saved in PIT entry for a chunk (Wang & Bensaou, 2012a);
- Node-Connectivity: one-hop neighbor nodes (Zhang et al., 2016);
- Node-Resource: number of interfaces (Wang & Bensaou, 2012a; Baugh & Guo, 2018).

Although the node’s location is a context that should be considered when selecting a replacement policy, it is not easy to foresee a straight map between policies and node positions. First, because there are many policies and diversity of topologies with different requirements, but mostly because there are other contextual factors that can also impact the performance of the policies. As we continue to show in the next sections, this SLR was able to pinpoint some of these factors.

#### Node: cache size

The works from Chao et al. (2013), Wang et al. (2014a), Sun et al. (2014), Newberry & Zhang (2019) and Liu et al. (2019b) contains evidence of cache size variations on the performance ranking variations of cache replacement policies. Table 8 summarizes the characteristics of the corresponding scenarios. In the following, we discuss the reported impacts:

- According to Sun et al. (2014), the replacement scheme’s optimal choice depends on the cache size and the placement policy. The authors combined seven placement policies with five replacement policies: LRU, LFU, FIFO, TTL, Size - and cache size variations of 0.0007%, 0.007%, 0.07%, and 0.7% of the unique contents. The content routers have homogeneous cache sizes for all experiments. We observe that the most significant impact on the replacement scheme choice happens when passing from 0.0007% to 0.007% of cache sizes. That is, for all combinations of placement policies, the best choice of replacement scheme changed when the cache size moved from 0.0007% to 0.007%. Meanwhile, for most combinations of placement policies, the experiments running with 0.007%, 0.07%, and 0.7% of cache sizes presented their highest performance values with the same replacement policy. For example, combined with LCE, LRU and TTL achieved the highest performances for 0.007% of cache size, while LFU stands out for the other sizes.
- Chao et al. (2013) also show evidence that variations on cache size can lead to variations on the policy with the best performance. This work presents a content-based replacement policy named FCDC that manages the content popularity property—request count—to classify and replace contents according to popularity categories. The evaluation shows comparisons of the proposed scheme against LRU and RUF policies. According to the results, FCDC presents a better cache hit rate than LRU and RUF when the cache memory is less than 5%. Yet, the performance rank changed for cache sizes larger than 10%, and LRU performed slightly better than FCDC. The authors attribute this behavior to each policy’s property, in which FCDC can keep track of content popularity and maintain the most popular content better than LRU for small cache sizes. At the same time, LRU prioritizes most recently accessed over the most accessed and popular content. However, this does not directly correlate to the performance differences according to the cache sizes. FCDC deals with dynamic changes of content popularity and does not directly rely on node information.
- Furthermore, the experiments performed by Wang et al. (2014a) also reveal differences in policy performance rank while varying the cache size. The work proposes the EV policy, a node-based replacement scheme coupled with a placement scheme. EV was evaluated and compared against LCE + LRU and LCE + Popu—a referenced popularity-based policy. The configuration of the content popularity follows a Zipf distribution, and besides the impact of different cache sizes, the results also reveal a correlation with the popularity skewness factor. For α skewness factor equals 0.8, EV and Popu had similar performances for all cache size variations. Meanwhile, for α = 1.5 or 2.0, the policies interchanged positions in the rank of average total energy consumption for different cache sizes: Popu achieved better performance than EV for cache sizes between 10% and 20% of total contents; for larger cache sizes, EV turns to be the better choice. The work does not provide an analysis of this effect. The results show the impact of cache size on placement and replacement schemes together, which limits the evidence of the eviction scheme solely.
- Similarly, Liu et al. (2019b) presented evidences of variations in the rank of hit ratio of the policies for different cache sizes. The work shows evaluations of a proposed replacement policy named PBRS against LRU, LFU, and FIFO. PBRS and LFU interchange positions for different cache sizes in a tree topology. This effect is most evident for intermediate nodes, in which LFU presented better results for cache sizes between 10 MB to approximately 50 MB, and PBRS presented better cache hit values for larger cache sizes. Both policies rely on content popularity, but LFU computes the popularity directly to count the number of requests, while PBRS increments the computation by adding different weights associated with the nodes.
- Finally, besides the effect of heterogeneous policies for different node locations in a fat-tree topology observed by Newberry & Zhang (2019), we also observed variations in policy performances’ rank while varying cache sizes. The work evaluated LRU and other replacement policies named 2Q, ARQ, LIRS, and MQ policies. For a homogeneous policy configuration in all levels of the topology, the rank of policy performances did not change when using cache sizes from 64 to 512 MB. However, when cache sizes varied from 512 MB to 1 GB, a couple of changes happened in the rank: first, LRU and 2Q interchanged positions, in witch 2Q achieved best results than LRU up to 512 MB, but LRU presented better results for 1 GB; second, ARQ and MQ changed positions, with MQ presenting better results up to 512 MB and ARQ with 1 GB; and finally, LIRS and ARQ also changed positions in the rank, with LIRS presenting better results than all other policies up to 512 MB, but ARQ achieved better performance with 1 GB of cache size. For a heterogeneous policy configuration, the results presented similar effects on the rank. Without going into specific characteristics of policies, this work has evidence of the influence of cache size and the lack of explicit patterns that associate the performance of cache policies with the size of the cache.

Regarding the impact on the replacement policy choice, in none of the presented works it is evident why variations in cache size led to different policy choices. Also, the analysis of the works together does not reveal potential patterns due to the heterogeneity of the scenarios factors. The scenarios range from country-wide router-level topology with around 80K routers to a small and straightforward linear topology, with variations of placement and replacement policies, and different ranges of cache size evaluations. Although the evidence clearly shows the relevance of cache size in particular scenarios, it is not sufficiently conclusive the why.

Yet, we cataloged other effects concerning variations on the cache cache and the performance of the policies. It is natural to expect an increase in the cache size should increase the performance gain for any caching policy since there is more space to store contents. In practice, the constraints of memory access speed or node devices’ power will limit cache size. However, evidence shows that *caching policies’ performance gain is not linear to the cache size increase* (Han et al., 2014; Chen et al., 2014; Ong et al., 2014; Sun et al., 2014; Pires et al., 2018; Mangili, Martignon & Capone, 2013). In this way, adding cache resources on the network could not be the most suitable solution to improve the performance. The observed effect is because size allocation is a function of the content’s popularity distribution. For example, in scenarios with large amounts of non-popular content, the cache size may be small because the gain in caching is restrictive. On the contrary, for scenarios with a large amount of popular content, the benefits will be best achieved for larger cache sizes. In this way, balancing optimal cache size in terms of cost and effectiveness of policies shall be done considering the fluctuations in content popularity.

Another observed effect of the relationship between cache size and replacement policy gain is that *as the relative cache size increases, the performance difference among the techniques decreases* (Charpinel et al., 2016; Han et al., 2014; Nakayama, Ata & Oka, 2015; Bilal & Kang, 2017; Xing et al., 2017; Panigrahi et al., 2014; Li, Simon & Gravey, 2012; Fricker et al., 2012b; Newberry & Zhang, 2019). That means that the performances tend to converge eventually, and this is in line with Che’s approximation (Che, Tung & Wang, 2002), which we briefly discuss here. The longest possible time between two sequential hits for a content *c* present in the cache, that is, before removing *c* from the cache, is expected to be random and related to *c*. That is the *cache eviction time* for content *c*. However, Che’s approximation stands that, for reasonably large cache sizes, this cache eviction time tends do be deterministic to the point of being a constant irrespective of the content. Therefore, as cache size increases, the dependance on *c* decreases and becomes negligible. Following this direction, if the dependance on the content decreases, the eviction policy’s dependance decreases because all contents converge to the same relevance in terms of eviction time. Although Che’s approximation has been proposed for a scenario with LRU under Independent Reference Model (IRM), other extensions and generalizations also show the approximation’s validity to more scenarios (Garetto, Leonardi & Martina, 2016; Fricker, Robert & Roberts, 2012a; Araldo, Rossi & Martignon, 2015).

#### Cache placement policy

ICN in-path cache works as an opportunistic cache to distribute the content along with the network, and that opportunistic characteristic makes more flexible the distribution of caches on network nodes and the content location choices. Once there is a cache, though, the replacement scheme is mandatory for all cache nodes. Nevertheless, both content placement and replacement decisions are closely correlated and influence each other behaviors. The decisions can be implemented separately and combined according to the network requirements. Each combination of placement and replacement policies can lead to different behaviors.

On the other hand, both placement and replacement strategies may complement each other. Some of the replacement schemes reported in ICN literature are already coupled with a placement strategy (Neves dos Santos et al., 2013; Sinky et al., 2018; Ren et al., 2014; Hu et al., 2015; Pal & Kant, 2017; Xing et al., 2017; Mick, Tourani & Misra, 2016; Zhang, Tan & Li, 2017b; Wang et al., 2014a; Chen et al., 2017; Khan & Khan, 2017) and deployed in conjunction.

In this work, we chose to look at the placement policy as a context factor that influences the replacement policy choice. This subsection presents the works (Chen et al., 2016; Tarnoi et al., 2014; Sun et al., 2014) in which variations in the placement policies led to different choices of replacement schemes:

- Chen et al. (2016) develop an ICN WSN system in which they tested 16 combinations between four placement strategies: LCE, Prob (i.e., LCP), LCD, and Betw (i.e., LCB)—and four replacement policies: Random, FIFO, LFU, and LRU—in a WSN with 15 nodes. The results reveal a significant variation in the rank of policies for different combinations of placement policies and comparison metrics. Considering the metric cache hit rate, LCE and Prob achieved their best results combined with LFU, while LCD and Betw with Random; Yet, when considering the metric energy consumption, LCE and Prob achieved their best results with FIFO, while LCD with LRU, and LCB with Random.
- In addition to analyzing the effect of heterogeneous policies configuration by node locations, Tarnoi et al. (2014) also analyzed variations on the replacement scheme choice according to the different placement policies. The work shows how the probabilistic caching placement behavior varies as a function of the replacement scheme. The authors evaluated combinations of LRU, LFU, and Random policies with LCE and Prob. In general, for both cascade and Internet-like topologies, and considering both server load and round trip-hop distance evaluation metrics, the results show that Prob can improve the performance of the network and achieve its best performance only when combined with LRU, while LCE achieves its best performance when in conjunction with LFU.
- Finally, as we mentioned earlier, the results reported by Sun et al. (2014) show that the optimal choice of the replacement scheme depends on the cache size and the placement policy. Regarding the variations of placement policies, the work combined seven placement policies: LCE, LCD, Rand, Prob, ProbCache, Betw (i.e., Cent), and CRCache (i.e., Cross)—with five replacement policies: LRU, LFU, FIFO, TTL, and Size—and the results presented evidence of the difference in performance ranks for each combination. For example, considering the metric server load reduction and 1G of cache size, LCE, Rand, Prob, and ProbCache achieved its highest values when combined with TTL; while LCD with FIFO; Betw with LRU; and CRCache with TTL or LRU. However, for cache sizes of 100G and 1T, all placement policies presented their best results with LFU, except for LCD, which achieved the best results combined with LRU or TTL. The work also stands for a dominant strategy among the compared ones in terms of caching metrics. Partially in line with Chen et al. (2016), and contrary to the analysis presented by Tarnoi et al. (2014), the authors place Prob + LFU as the closest to the best strategy for their scenario. However, the analysis between the different results is limited because the two works (Chen et al., 2016; Sun et al., 2014) did not mention the probability value used for caching contents. The Prob performance may vary according to the configured probability value.

Reinforcing the intrinsic correlation property between content placement and replacement decisions, all the works presented in this section show evidence of the different and unique effects of each policy’s combinations for distinct scenarios. Different placement policies can have a different impact when changing a replacement scheme (Rezazad & Tay, 2015; Tarnoi et al., 2015; Zhang et al., 2017a; Meddeb et al., 2017). This way, each placement strategy requires evaluation of what replacement scheme performs the better. Each placement policy has a different requirement in terms of evictions, and the more is the number of evictions, the more the placement policy relies on the replacement scheme and, therefore, is affected accordingly.

#### Content: popularity

One of the behaviors we were expecting to find evidence for was the impact of content popularity variation on the replacement policy choice, especially on the choice between frequency-based policies, for example, LFU, and others, like recency-based policies. That reasoning relies on the argument of many works that frequency-based policies suit better content populations with high popularity skewness, while with low popularity skewness would suit other policies (Beck et al., 2017).

However, while analyzing the variations of popularity skewness during the comparative evaluation of the replacement schemes, we found works in which *popularity skewness variations did not influence policies’ rank* (Wang et al., 2011; Gür, 2015; Huang et al., 2017; Zhang, Tan & Li, 2018; An & Luo, 2018; Jeon, Lee & Song, 2013; Shailendra et al., 2016; Liu et al., 2017; Tarnoi et al., 2014; Gallo et al., 2014; Yokota et al., 2016; Zhang, Tan & Li, 2017b; Sinky et al., 2018; Yao et al., 2016). Those comprehend works under Zipf popularity distribution, with different variations of the skew factor from, for example, 0–2, with conventional policies like LRU and LFU as well new proposed policies, but the performance rank among the policies remained unchanged. Variations in the skew factor represent variations in the distribution of contents’ popularity. The increase in the factor leads to an increase in the number of popular content. It is also associated with the diversity among contents. The increase in the number of popular contents reduces the diversity of the contents stored in the caches since popular contents are more conducive to occupy cache spaces for relatively long times.

Also, we observed a similar effect as the one about the increasing of cache size discussed earlier: under variations of the skew factor solely, *as the skew factor increases, the difference of performance among the techniques decreases* (Badov et al., 2014; Yokota et al., 2016; Zhang, Tan & Li, 2017b; Zhang, Tan & Li, 2018; Sinky et al., 2018; Yao et al., 2016). For instance, during the evaluation of a proposed PPC policy, Zhang, Tan & Li (2018) carried out experiments varying the Zipf skew factor from 0.7 to 1.2. They compared the policy performance against LRU, LFU, FIFO, and CCP. The results reveals that as the factor increases, the difference of cache hit ratio among the replacement schemes is reduced and tends to converge.

#### Remaining remarks

This section presented many scenarios with evaluations of cache replacement policies that presented different behaviors according to variations in contexts. Contextual factors are triggering this difference in performance, and this SLR was able to identify some common factors in a set of works, as we exposed in the previous subsections.

The influence of some contextual factors was already evident when looking at individual works. However, one of our intentions with this SLR was to analyze the works that had similar effects, to look for patterns that could relate the contextual factors to the policy’s properties. That cames in contrast with the diversity of scenario characteristics and evaluated policies, which limited the analysis. Besides, there was no more in-depth analysis of why and how the effects happened, most of the works came to evidence by testing the context variations, and small changes in the characteristics of the scenarios could have lead to different results. In general, there was no explicit pattern in the surveyed works that associated the context factor to the policies or their properties. That also limited a more in-depth analysis from the perspective of the proportion of impacts for different contexts and scenarios, since the extent to which context characteristics affected cache replacement strategies varied for different scenarios.

We must also highlight that most of the works did not indicate the confidence interval in their experiments. A few of the differences between policies’ performance measurements were relatively small, and a confidence interval would help investigate the significance of the difference values.

Due to the reasons mentioned above, the policy choosing process can not be reduced to rule-based schemes or related solutions. Instead, the choosing process is suitable for solutions that dynamically analyze context factors and perform large-scale correlations between the factors and policies, for example, with reinforcement learning techniques. At this point, we can indicate, though, potential context characteristics to enhance the eviction performance in emergent ICN scenarios. We present this analysis in the next section (Applications).

Lastly, we also highlight the overlook of content-Negative Acknowledgments (content-NACKs) packets. In ICNs, content-NACKs are special packets generated by content producers in response to requests for non-existent content. They can be encoded as data packets with a specific content-type feature. In that case, content-NACKs are processed as regular data packets and cached in the network routers. Although caching content-NACKs is useful to respond to possible subsequent requests for the same non-existent content efficiently, it may insert vulnerability points in ICN architectures (Compagno et al., 2015). Current eviction policies are not aware of content-NACK packets, and there is a need to investigate if this lack of awareness impacts cache management and security. Nonetheless, a different approach is questioning if those packets should be cached on the network and how it could impact performance. In deciding not to cache, the processing of content-NACKs can be delegated to the cache placement policies to bypass these packets. This way, the content-NACKs could follow the forward processing of data packets without caching.

## Applications

The informational RFC 7476 (Pentikousis et al., 2015) presented by the IRTF-ICNRG describes a set of application areas in which ICN architectures can potentially perform better than the current host-centric Internet approach. This technical document discusses diverse network contexts in emergent areas such as social networking, real-time communication, mobile networking, vehicular networking, delay- and Disruption-Tolerant Networking (DTN), IoT, and Smart Cities.

Thus, we extend the discussion to correlate characteristics of emergent networks with the context characteristics relevant to the choice of suitable cache replacement schemes. We highlight the most suitable context characteristics for generic network contexts on information-centric IoT (Arshad et al., 2018; Dong & Wang, 2016), vehicular named-data networking (Khelifi et al., 2020), and ICN-enable edge and core networks (Zhou et al., 2017; Zhang et al., 2018) in the following subsections. Table 9 summarizes this discussion.

**Table 9:** Suggestion of cache replacement policy category for different ICN-enable scenarios.

Cache-enable network	Characteristics and/or requirements	Policy category	Correlation of requirements with context dimensions
IoT (Smart home, home care…)	High heterogeneity among IoT devices with different priorities; High ephemerality of contents; Limited resources	Content and node-based	Content features, like content provider identification, priority, and time-related properties
VANETs	High intermittency of connections; Multi-path propagation; Different strategies for delay-sensitive data from safety applications and delay-tolerant data from infotainment applications	Content and node-based	Node location properties like mobility pattern plus direction, node’s rank according to topology position; Content features, like type, priority, and popularity and time-related properties
Edge computing (Small-cells radio access; 5G; Device-to-device (D2D) communication; Unmanned Aerial Vehicles (UAVs))	High temporal and spatial correlation of content requests; Enables clusters by user similarities	Content and future human-based	Content popularity properties; User preferences, habits, and social interaction
Internet-scale networks	Globally content preferences; Heterogeneous link/node capacities; Long geographical distances	Content, node and network-based	Content feature and popularity properties; Network topology, resource, and time-related properties; Node resource and traffic properties

### Information-centric internet of things

The adoption of IoT networks in many segments of society like healthcare, transportation, security, industry, agriculture, communications, and infotainment, is gradually changing the way people interact with the physical world by connecting new *things* to the Internet. Things can be any device enhanced with sensor and technology capabilities to generate and transmit data, and when aggregated with intelligent services of IoT applications, they can improve processes, business, and life quality.

The imminent revolution of IoT applications must be followed by a revolution in how the network structure deals with the *content*. The current Internet architecture is fundamentally not prepared to deal with the massive amount of data from an expected number of billions of heterogeneous devices. The majority of IoT applications will be content-oriented, and TCP/IP will be struggling to meet their bandwidth requirements. Cache-enabled solutions like information-centric architectures are strong candidates to assist in the deployment of IoT applications (Arshad et al., 2018; Quevedo, Corujo & Aguiar, 2014; Dong & Wang, 2016; Araújo, De Sousa & Sampaio, 2019). The ubiquitous content caching of ICN contributes to reducing the delay to retrieve content and enhances the contents’ availability, especially when dealing with power restricted devices that periodically switch on and off in duty cycling to save resources.

In cache-enabled network solutions, IoT traffic usually is offloaded at the Internet content routers through a connected gateway (Rao, Schelen & Lindgren, 2016; Meddeb et al., 2017) to aggregate the services of specialized IoT cloud platforms, such as Cisco IoT Cloud Connect, Microsoft Azure IoT Suite, and Google Could IoT. Also, the IoT devices can cache the traffic in a dynamically distributed IoT network (Hahm et al., 2016). Whether one case or another, two significant characteristics are a large number of heterogeneous devices and the ephemerality of the content produced by them. Therefore, the suitable kind of cache replacement schemes for information-centric IoTs should deal with both characteristics. In the former, the different types of devices usually have different resources restrictions in terms of processing capabilities, memory, energy constraints, and they produce contents with different requirements regarding the context. For example, Smart Cities will need to integrate intelligent urban sensing services for many proposes, such as management of smart garbage collection, street lighting, parking, the monitoring of road conditions, urban noise, security cameras, and environmental conditions, among other possibilities. In this case, the infrastructure comprises a diversity of sensors with different content production rates and characteristics. The replacement scheme may apply different treatment to the contents according to the type of device by exploring both *content* and *node context dimensions*, with features like *content provider identification*, *content priority*, and *node resource features*. The latter characteristic points out the typical time-restricted data generated by some IoT devices that periodically inform sensor measurements monitoring the environment. For example, the content periodically generated by temperature sensors and collected by distributed applications to monitor the ambient in urban areas can be usefully cached to serve user applications’ requests. However, the most recent measure will usually be of interest to most applications, and there is no need to maintain the previous measures in the cache. The replacement scheme should also combine *time-related features* of the *content context dimension* in the eviction process logic. The combinations of the features mentioned above can help detect redundant contents from the same producer while increasing the techniques for stale content detection.

### Vehicular named-data networking

Vehicular networking exhibit singular characteristics in traffic generation patterns, delivery requirements, and spatial and temporal scope (Pentikousis et al., 2015), mostly due to high node mobility, very intermittent connections, and the support for typical road-traffic-related applications (Li et al., 2020a), infotainment applications, and code dissemination (Li, Zhao & Wong, 2020b).

In vehicular networking, the vehicles can exchange information with any other communication device available next to the vehicle in a concept of Vehicle-to-everything (V2X) communication. This includes communication between vehicle and other vehicles (Vehicle-to-Vehicle—V2V), or road infrastructure (Vehicle-to-Infrastructure—V2I), communication network structure (Vehicle-to-Network—V2N), pedestrians (Vehicle-to-Pedestrian—V2P), or any other communication device. In all those variations, the content requests usually present highly temporal/spatial dependencies, and the in-network caching capabilities of ICNs can potentially improve the content delivery process.

Regarding the caching strategy, the replacement scheme should consider the characteristics mentioned above because they can affect the local relevance of contents. For example, accident information’s relevance is highly dependent on the vehicle location and the direction towards it was moving (De Sousa, Araújo & Sampaio, 2018). If the vehicle has passed the accident, that information may no longer be useful. The replacement schemes can handle this decision with *node location properties* like *mobility pattern*, plus *vehicle direction*, and *node’s rank according to the current topology position*.

Different strategies should be applied to deal with the different types of applications combined with node location properties. For that, the strategy can explore *content features*, like *content type* and *content priority*. The road-traffic-related applications, such as road congestion notification, traffic monitoring, and accident warning, usually are delay-sensitive applications and are better handled by *content time-related properties* or even newly *type-specific properties*. Similarly, applications for code dissemination designed to support smart city infrastructures’ upgrades can benefit from those properties. Meanwhile, the infotainment applications are mostly delay-tolerant and more suitable to be handled by *content popularity* features.

### In-network cache-based data offloading through edge computing

Caching at the edge in Mobile Edge Computing (MEC) (Safavat, Naveen & Rawat, 2019) will play an essential role in the next-generation wireless network. The Radio Access Network (RAN) is enhanced with cache capacity on base station structures to better attend the content demand due to its proximity. This way, Small-cell Base Stations (SBS), Macro-cell Base Stations (MBS), Wi-fi Access Points (AP), mobile devices, and even recent cache-enabled UAVs (Zhang et al., 2020; Ji et al., 2020; Huang et al., 2020) can store contents and respond to the content requests faster. UAVs can act as flying base stations to support the ground cellular network. They can also work as relay nodes to assist content delivery and data collection in areas without available transmission links. The integration with ICN concepts leverages the mobile-edge caching by supporting in-network caching (Zhou et al., 2017; Psaras et al., 2018; Shariat, Tizghadam & Leon-Garcia, 2016). The imminent fifth-Generation (5G) mobile networks also reinforces that merge as several initiatives discuss the benefit of the integration with ICN (Zhang et al., 2018; Liang, Yu & Zhang, 2015).

A fundamental characteristic created by the user’s closeness is a high temporal and spatial correlation of content requests. In this way, one of the widely explored approaches at the network edge is user-centric clustering techniques (Ribeiro, Sampaio & Ziviani, 2018; He, Wang & Wang, 2019; ElBamby et al., 2014). User characteristics are the input and motivation for virtual groupings, whether regarding the network structure or the users’ connection to the network. As a consequence, user and their content requests can be grouped according to user behavior patterns.

Due to the characteristics above, the replacement schemes for in-network caching at the edge can benefit from *content-based properties*, especially *content popularity* features, and the exploration of a variety of *human properties* related to preferences, habits, and social interaction. Therefore, user behavior analysis is a relevant area in the future of edge-caching, fostering future human-based replacement policies.

### ICN-enabled core network

ICN’s benefits encompass large-scale networks with backbone core nodes and high-speed links with different capacities, interconnecting heterogeneous Autonomous Systems (AS) with multiple access networks. In this way, core networks aggregate content requests from different access networks, and unlike the edge, the temporal/spatial correlation of requests is gradually reduced and becomes weaker as the content requests approach the core nodes. Many solutions enhance ICN’s applicability at core network structures for inter-domain network services such as routing (Liu et al., 2019a), traffic engineering (Li et al., 2019), and globally accessible name schemes (Van Adrichem & Kuipers, 2013).

Because of the considerable physical distances naturally presented in large-scale networks to connect content consumers and producers, requests typically have to traverse several nodes within the network. Therefore, the *network topology* context must be taken into account to optimize cache replacement policies in content-based core nodes. Context properties, in this case, are related to the *distance* connecting two end-nodes, like *hop count*, properties related to the *network resources*, like *packet transmission cost, link capacity*, and *time-related features* with *network delay for retrieving content*.

The cache replacement schemes should also explore *content* and *node* contexts to reflect globally content preferences and the different capacities of core nodes, respectively. The *content feature* and *popularity properties* and *node resource* and *traffic properties* may further increment the replacement policies’ decision. On the other hand, there is a trade-off relating the performance while processing many context information, since core routers process requests at line speed.

## Research directions

In this section, we discuss different research directions for context-aware cache replacement schemes in ICNs.

### Context information management

Dealing with contextual information requires well-defined procedures on acquiring, representing, reason, and distributing the information. Context information management is widely studied and applied in many sciences that rely on context-awareness (Perera et al., 2013). Still, it is a challenge for complex systems such as dynamically distributed networks to efficiently perform online context management, especially when there is a need to represent a high number of dimensions and elements relevant to represent the domain. The integration between ICN and SDNs (Kim, Chung & Moon, 2015; Charpinel et al., 2016; Yao et al., 2016; Kalghoum, Gammar & Saidane, 2018; Liu et al., 2018; Saadeh et al., 2019) can further benefit context management solutions because of the SDN paradigm’s centralized control view. It is necessary to investigate what context information could be efficiently handled by central controlling.

The sets of context features identified within our proposed classification are enablers to a semantic representation of the context domain and can be extended or adapted according to different application requirements. However, towards an efficient real-world deployment, there is also the need to argue about the *quality* of context information. Quality can associate many aspects like reliability, precision, timeless, access right, significance, granularity, and completeness. Those aspects are translated into metrics defined by the science of Quality of Context (QoC) (Buchholz, Küpper & Schiffers, 2003). The relevance of QoC metrics varies following the type of information. Hence, different QoC metrics should follow the different context subcategories in each context dimension.

### Scalability of context suitability

Exploring context information is essential to address a mismatch between caching policies and emerging networks. This exploration contributes to achieving more potentially precise and customized techniques. However, the more the use of contextual information, the more computationally expensive the caching scheme might become. The need to compute more context information may increase the complexity of the caching policy itself. Therefore, it is essential to investigate the *performance cost* of individual context information and the solution as a whole. The performance cost depends not only on managing the information but also on how the policy treats the information.

### Machine learning techniques

In addition to being used for context information inference (Zhao et al., 2017; Nakayama, Ata & Oka, 2015; Liu et al., 2018), machine learning techniques can investigate how to exploit better context information to optimize the eviction process.

In one perspective, machine learning techniques could select which contextual information is most relevant and should shape the eviction process. The relevance of contextual information may vary depending on the network and objectives. This way, given a network with a set of available contextual information, it would help investigate how to choose what should be used by the eviction scheme to increase network performance.

In another perspective, the techniques can direct the learning of the best kind of policy based on what context information is available. Reinforcement learning techniques have been successfully applied for caching schemes (Sung et al., 2016; Sadeghi, Sheikholeslami & Giannakis, 2017). However, in those works, the context state is represented solely by the cached contents in an instant of time. It would be relevant to extend the concept of context to represent the state with more available information that would impact the learning policy process. Depending on the number of context information used, there may be a large space of possible states, which will require considerable computational effort to represent the possible variations. When most of the states are rarely revisited, the chosen technique must deal with some sort of generalization. Furthermore, model-free techniques are best indicated when there is no previous knowledge dataset to help the decision process.

### Dynamic and adaptive instantiation of cache policies

Along with SDN and ICN, Network Function Virtualization (NFV) techniques are strong candidates for realizing and fostering next-generation networks (Zhang et al., 2018; Saadeh et al., 2019). Through the network function virtualization concept, in-network caching strategies can quickly execute as Virtual Network Function (VNF) along with some management structure. This combination paves the way for efficient deployment of adaptive caching policies according to the context’s dynamic changes. To realize a plug-and-play vision of virtual function would be interesting to have a rich repository of heterogeneous caching functions and multi-attribute functions exploring different combinations of context information.

### Human aspects

In recent years, the community has witnessed a growing number of researches focused on solutions that exploit the human-user context to solve problems in different areas (Shafigh et al., 2019; Zaidi et al., 2019; Zeng et al., 2019). Due to mobile computing expansion, networking-related studies also tend to consider human aspects such as interactions, social ties, and personality to propose human-awareness solutions. This movement from device-to-device to people-to-people communication paradigm aims to look at network configurations taking into account the user’s perspective, integrating human perception approaches with QoS metrics, and further, with the mapping of user behavioral profiles. Network contexts are more likely to cope with group-based rather than individual user profiles. Different user profiles, such as personality profiles, may reflect distinct patterns of how users in each profile interact with the network, and consequently, each profile may produce different impacts on the network resource consumption. Therefore, the network can adapt according to the predominant user profiles to improve the distribution/consumption of resources and user QoE at the same time.

In ICN research, human factors present great potentials to improve the communication service delivery, in particular through adaptive caching solutions (Ribeiro, Sampaio & Ziviani, 2018). One approach is to explore potential correlations between user characteristics and cache policies and adopt mechanisms for dynamically adapt the most suitable caching strategies to the predominant user behavior. A key challenging consists of finding out the human aspects that most positively impact the network efficiency and how they could be operatively explored in ICN architectures. That requires a multidisciplinary view with the integration of psychology research to support lower granularity levels of user information.

### Privacy

In-network cache aggregates benefits to ICN architectures by reducing bandwidth consumption and the latency to deliver contents over the network, but it also introduces architectural vulnerabilities regarding cache privacy (Acs et al., 2013). For example, in side-channel timing attacks, a malicious user can deduce what content was accessed recently by another user on the same network by merely measuring content delivery times with standard content requests. Acs et al. (2013) discussed techniques for mitigating privacy caching attacks in which contents marked as private could have different treatments by the cache management mechanism. One countermeasure presented to inhibit the timing attack consists of the insertion of artificial delay times in the content delivery process, so the malicious user cannot differentiate which content was retrieved from the cache or directly from the producer.

Recent efforts from the NDN research community have tried to address many of the current privacy concerns (Compagno et al., 2020; Dogruluk et al., 2020), but more work lies ahead concerning the context information processed by caching strategies. The use of context information to allow the dynamic adoption of the most appropriate cache policy may require the processing of sensitive data of related users stored in communication devices. One major concern resides in guaranteeing the anonymity of data processed, particularly involving users for privacy-preserving cache management.

Similarly, there is a concern about the privacy of cache management strategies adopted on the network routers. Fan et al. (2020) recently presented a method capable of detecting the placement policy configured in the routers. As described in the malicious attempt to discover the previously accessed content in the network, the method does not require any privileged access and can infer a placement policy through ordinary content requests. Knowing the strategies used for content management can enhance the inference mechanisms of accessed content.

## Conclusions

This article presented a comprehensive and systematic review of studies regarding cache replacement policies in ICNs. The literature presents a vast set of eviction strategies exploiting combinations of multi-dimension aspects of context information in different ways, aiming at making more customized and effective decisions about the relevance of contents. Thus, among its findings, the SLR showed the relevance of considering context’s properties in choosing suitable replacement policies. The study revealed that efficient utilization of cache resources in ICNs relies on deploying cache replacement policies according to the network contexts. The SLR contributes to characterize the context factors correlated with the caching policies and the reported effect of context variations on cache replacement policies’ performance. The compilation of evidence shows no single context factor determining the choice of policies; there is no explicit pattern regarding context properties variations to support the choosing process of policies for different network contexts. The results reaffirm the absence of a single optimal strategy to meet the requirements of all network since the caching policies’ performances vary according to different context characteristics. Additionally, the dynamic nature of most networks leads to on-demand changes in the context characteristics, for instance, changes in traffic patterns or user preferences, and the ICN strategies must adapt to these changes in an attempt to ensure the best network performance. Therefore, there is the need to assist the choosing process of suitable schemes according to the current context, and further, to cope with the natural dynamism of context variations in networks.

## Additional Information and Declarations

### Data Availability

The following information was supplied regarding data availability: The study did not use raw data or code as it is a literature review.
